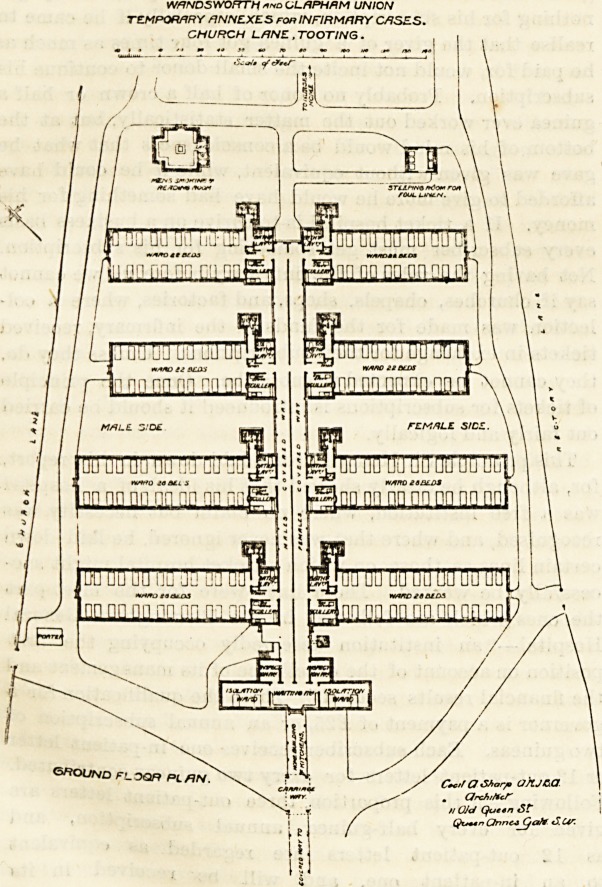# Temporary Hospital Wards at the Wandsworth and Clapham Union

**Published:** 1903-04-04

**Authors:** 


### HOSPITAL ADMINISTRATION.

### CONSTRUCTION AND ECONOMICS.

### TEMPORARY HOSPITAL WARDS AT THE WANDSWORTH AND CLAPHAM UNION.

THE only ground owned by the Guardians at Church Lane, Tooting, on which these blocks could be erected was a piece lying between the main building and the Nurses Home. This plot tapers towards the north, and it required some ingenuity on the part of the architect to fit the various blocks on it, as not only had the bye-laws to be borne in mind, but the proximity of the permanent buildings had also to be kept in view. We think the architect has succeeded in his somewhat difficult task. The land available for his purpose has been divided lengthways by a double corridor, one side of which is the connecting medium with the men's wards and the other with the women's. At the south end of this double corridor are the wards for infectious cases, and a covered way commencing at this point joins the new temporary wards with the older permanent buildings.

A glance at the accompanying plan will show the arrangement of the blocks; and as these blocks and the double corridor are only one story high, no objection can be raised to the arrangement, or to the nearness of one block to its neighbour. Indeed, bearing in mind the kind of cases to be treated in the hospital, the arrangement is decidedly good, and it is very compact.

There is accommodation for 204 patients—102 of each sex. Altogether there are four wards for 28 beds each, and four for 22 beds each, and two isolation wards for two beds each. The blocks are intended for the ordinary sick inmates from the Wandsworth and Clapham Union.

The 28-bedded wards are 84 feet long, 24 feet wide, and 11 feet high, and so give about 63 superficial feet and about 800 cubic feet per bed. This may be quite sufficient for the class of patients to be treated; but, of course, it is much less than would be considered necessary in a general hospital. It will be seen from the accompanying plans that the beds are arranged in pairs, each pair having a window on each side. We are aware that this arrangement is sometimes adopted, but it is one of which we cannot approve; and even although the wall space is only 6 feet per bed, we should prefer to have seen a window on each side of each bed. It is true that the windows would have been narrow and the elevation somewhat spoiled by the number of windows, but mere appearance need not be too much considered in the construction of a hospital.

At the corridor end of each ward there is a scullery in one corner and a bath-room in the other. The sanitary annexes project beyond the bath-rooms, and they are properly cut off by cross-ventilated passages.

There is a small block near the north-east corner of the ground used as a smoking and reading-room for the men. Seventeen additional nurses are required, and rooms for these had to be provided in the permanent building.

The food is cooked in the main kitchen, and is conveyed to the blocks along the covered way already mentioned.

The walls of the blocks are constructed of timber framing covered externally with corrugated iron. The roofs are also of iron. The inner sides of the walls are plastered on steel laths.

The wards are warmed by hot-water radiators, the boiler being conveniently placed under the isolation block. No open fireplaces are provided. This is a great oversight, as, however useful hot-water radiators may be in exceptional circumstances they should never be the sole, and rarely the chief, means of warming an infirmary. The sanitary fittings are of porcelain, and a perfect system of drainage has been carried out.

The architect is Mr. Cecil Sharp of Old Queen Street, S.W.

The cost of the buildings was £15,000, but this sum included the additions to the permanent building.

## Figures and Tables

**Figure f1:**